# Primary Elbow Arthroplasty in the Management of Complex Distal Humerus Fractures

**DOI:** 10.7759/cureus.65851

**Published:** 2024-07-31

**Authors:** Uday D Mahajan, Kashif Memon, Sonu Mehta, Samuel Chan, Edward Spurrier, Socrates Kalogrianitis

**Affiliations:** 1 Trauma and Orthopedics, Queen Elizabeth Hospital Birmingham, Birmingham, GBR; 2 Trauma and Orthopedics, Glan Clwyd Hospital, Rhyl, GBR

**Keywords:** patient selection, postoperative management, functional outcomes, distal humerus fractures, primary elbow arthroplasty

## Abstract

Introduction

Complex distal humerus fractures pose significant challenges in orthopedic surgery, especially when traditional open reduction and internal fixation (ORIF) is not feasible. Primary elbow arthroplasty has emerged as an alternative treatment option for these fractures, but its application remains limited. This study aimed to evaluate the functional outcomes, patient selection criteria, and follow-up results of primary elbow arthroplasty in the management of complex distal humerus fractures.

Methods

A retrospective review was conducted on 15 patients who underwent primary elbow arthroplasty for Orthopaedic Trauma Association (OTA) type C distal humerus fractures between 2017 and 2023 at our institution. Inclusion criteria were patients aged 18 years or older who were offered either total elbow or hemiarthroplasty for acute complex distal humerus fracture. Data were collected from patient medical records, including demographic information, fracture classification, surgical details, and postoperative follow-up. Functional outcomes were assessed using the Oxford Elbow Score (OES) and Mayo Elbow Performance Score (MEPS). Complications were documented, and descriptive statistics were used to summarise the findings.

Results

The mean age of the patients was 71.8 years (IQR 17 years), with 12 females and three males. The mean time to surgery was 14.7 days post-injury (IQR: 12 days). The mean follow-up duration was 52 weeks (range: 8-234 weeks, IQR: 27 weeks) and variability was noted. The mean flexion-extension arc at the final follow-up was 93° (IQR: 32.5°). The mean OES was 46 (IQR: 22), and the mean MEPS was 75 (IQR: 37), indicating good to excellent functional outcomes. Scores for two patients were not available due to dementia. Reported complications included one case of ulnar sensory symptoms and one case requiring metalwork removal following olecranon osteotomy.

Conclusion

Primary elbow arthroplasty provides a viable treatment option for complex distal humerus fractures, demonstrating significant functional improvements and high patient satisfaction. However, the variability in follow-up and subjective decision-making underscores the need for standardized protocols. Future multicenter, prospective studies with larger cohorts and standardized follow-up protocols are recommended to confirm these findings and optimize patient care.

## Introduction

Elbow fractures, particularly those involving the distal humerus, pose significant challenges in orthopedic surgery due to the complex anatomy of the elbow joint, osteoporosis, and the frequent involvement of multiple bone fragments [1]. Distal humerus fractures, classified as OTA type C, are characterized by their complexity and are subdivided into three categories based on the extent of articular and metaphyseal fragmentation. These fractures often necessitate intricate surgical interventions to achieve stable fixation and restore function [2].

Gold standard methods of fixation, such as open reduction and internal fixation (ORIF), often face limitations in these cases due to the difficulty in achieving stable fixation, particularly in osteoporotic bones and highly comminuted fractures. There is a high risk of complications such as nonunion, malunion, and posttraumatic stiffness [3]. Primary arthroplasty has emerged as a viable treatment option for managing complex elbow fractures that are not amenable to conventional fixation techniques [4,5]. This approach involves the replacement of the damaged elbow joint with an artificial prosthesis, providing immediate stability and facilitating early mobilization. While primary elbow arthroplasty is well-established for rheumatoid joint diseases, its application in acute fracture settings has gained recognition more recently [6]. Several reports have documented its efficacy, and it is now acknowledged as a treatment option for specific cases of elbow fractures [7].

Despite this recognition, primary elbow arthroplasty remains a relatively infrequent procedure. For instance, in 2022, only 429 elbow replacements were performed for fractures in the United Kingdom, highlighting its limited but growing utilization [8]. The existing literature, while valuable, reflects a relatively small number of cases, underscoring the need for more data to better understand the outcomes and refine the indications for this procedure.

Our focus in this study is on functional outcomes, patient selection, and follow-up of patients undergoing primary arthroplasty for complex elbow fractures. By evaluating these aspects, we aimed to contribute to the existing body of literature and provide insights that can help optimize treatment protocols and patient selection criteria. Through a comprehensive analysis of our outcomes, we hope to enhance the understanding of the benefits and challenges associated with primary elbow arthroplasty in the management of complex fractures.

## Materials and methods

This study was designed as a retrospective review of patients who underwent primary elbow arthroplasty for the treatment of complex distal humerus fractures classified as OTA type C. Data were collected from patient medical records, including demographic information, fracture classification, details of the surgical procedure, and postoperative follow-up information. Functional outcomes were assessed using the Oxford Elbow Score and Mayo Elbow Performance Score, and complications were documented. Patients included in the study were those who presented to the emergency department (ED) with complex elbow fractures and subsequently underwent primary elbow arthroplasty between 2017 and 2023 at our institution.

The inclusion criteria comprised adult patients diagnosed with complex distal humerus fractures (OTA type C) that were unsuitable for internal fixation and required primary elbow arthroplasty as the initial treatment. Patients were excluded if they had open fractures or infection at the fracture site, were unfit for surgery, or had a history of previous elbow fractures. For the purposes of this study, arthroplasty is defined as a surgical intervention aimed at replacing the damaged elbow joint, which includes either distal humerus hemiarthroplasty (DHH) or total elbow arthroplasty (TEA).

Patients with complex elbow fractures were initially assessed and managed in the ED following the principles of the Advanced Trauma Life Support (ATLS) protocol. Standard imaging techniques, including X-rays and CT scans, were performed to evaluate the fracture pattern. All cases were discussed in a multidisciplinary trauma meeting held after the initial presentation. Considering the complex fracture pattern, patient physiology, overall health status, and preferences, the decision to proceed with primary elbow arthroplasty was made collaboratively by the trauma and orthopedic teams.

All surgeries were performed by experienced orthopedic surgeons specialized in upper extremity surgery. Patients were positioned laterally with the affected side uppermost. A participial approach was used in all cases, except for two total elbow arthroplasties where the triceps tongue approach was employed. An olecranon osteotomy was performed in one patient initially planned for ORIF, who presented with an olecranon fracture in addition to a complex distal humerus fracture. The Latitude prosthesis (Mahwah, NJ: Stryker Corporation) was used in all cases except one, where a Biomet prosthesis (Warsaw, IN: Zimmer Biomet) was used. All components were cemented. Postoperatively, all patients were encouraged to start with range of motion (ROM) exercises.

The primary outcomes measured were the range of motion (flexion, extension), the Oxford Elbow Score, and the Mayo Elbow Performance Score at the final follow-up. Documented postoperative complications, including infections, implant loosening, nerve injuries, and other relevant adverse events.

Patients were planned for follow-up at regular intervals as follows: six weeks, three months, six months, one year, and annually thereafter. During these visits, functional assessments and radiographic examinations were performed to monitor the integrity of the implant and detect any complications. Descriptive statistics were used to summarize patient demographics and baseline characteristics. Continuous variables were expressed as interquartile range (IQR) and categorical variables as frequencies.

## Results

**Figure 1:**
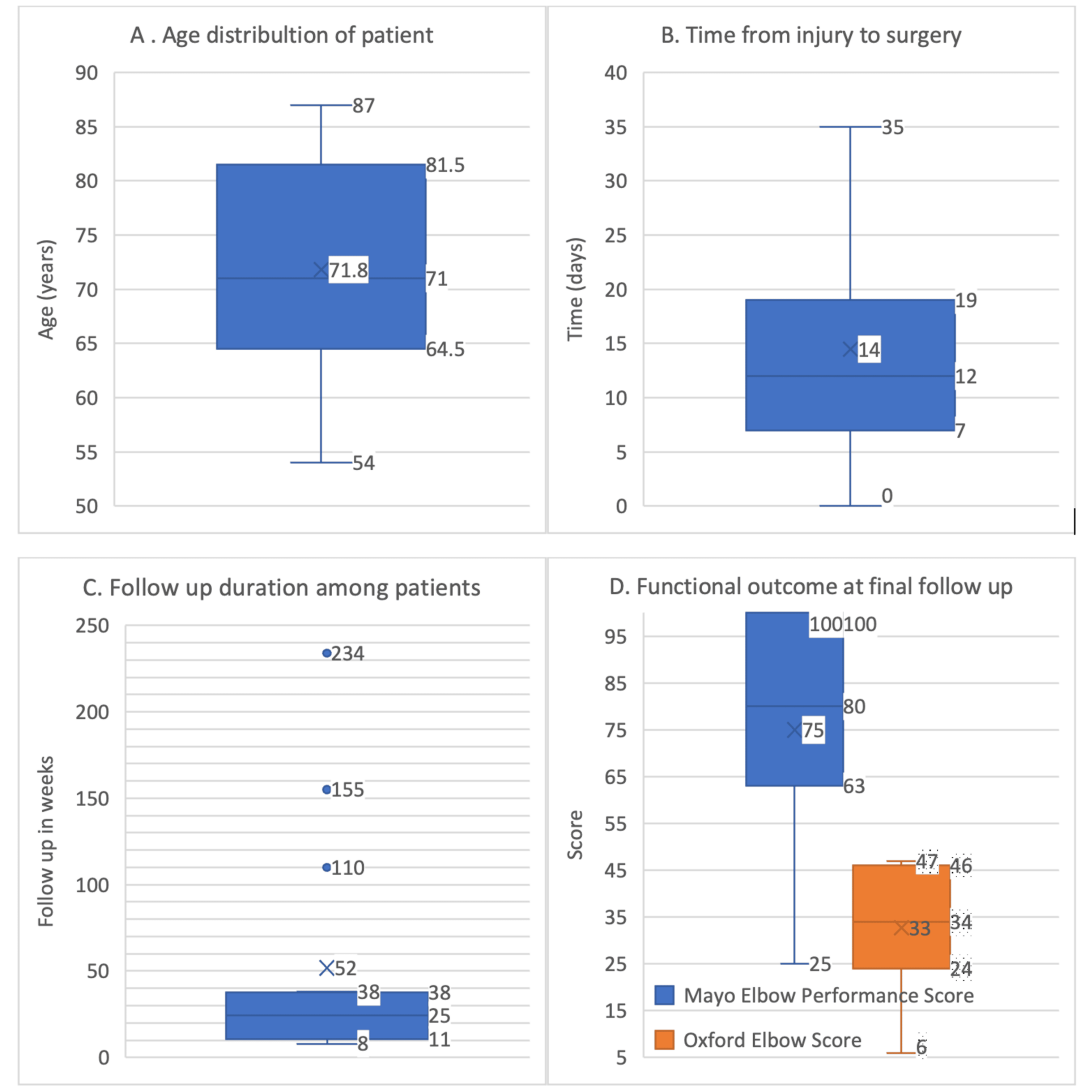
Demographic and clinical outcomes of patients undergoing primary elbow arthroplasty. This combined figure presents key demographic and clinical outcomes of patients undergoing primary elbow arthroplasty for complex distal humerus fractures. Panels illustrate the age distribution of patients (A), the time from injury to surgery (B), follow-up duration (C), and functional outcomes measured by OES and MEPS at the final follow-up (D). OES: Oxford Elbow Score; MEPS: Mayo Elbow Performance Score

This retrospective study, conducted between 2017 and 2023, included a total of 15 patients who underwent primary elbow arthroplasty for complex distal humerus fractures (OTA type C). The cohort comprised 10 patients who received DHH and five patients who underwent TER. The mean age of the patients was 71.8 years, with an interquartile range (IQR) of 17 years. There were 12 females and three males (Table 1 and Figures 1A-1D).

**Table 1:** Patient demographics and clinical data. All fractures were classified using OTA classification. POD: postoperative day; CKD: chronic kidney disease; Ca: cancer; RA: rheumatic arthritis; GORD: gastro-oesophageal reflux disease; OTA: Orthopedic Trauma Association; SAH: subarachnoid hemorrhage; T2DM: type 2 diabetes mellitus

Patient	Age (years)	Gender	Fracture type	Side	Comorbidities	Time between injury to surgery (days)	Discharge from hospital
1	62	F	Type C	Right	Osteoporosis, brain aneurysm	6	POD 3
2	65	F	Type C	Left	Asthma, osteoarthritis	32	POD 8
3	72	F	Type C	Right	Hypertension, asthma	4	POD3
4	85	F	Type C	Left	Aortic stenosis, CKD, mitral stenosis	12	POD 12
5	67	F	Type C	Left	HTN	11	POD 7
6	85	F	Type C	Right	Osteoporosis	8	POD 6
7	80	M	Type C	Right	Dementia, hyperlipidemia, SAH	3	POD 3
8	71	F	Type C	Left	Dementia, RA, T2DM	14	POD 3
9	54	M	Type C	Right	Asthma, bowel resection, ileostomy	35	POD 26
10	64	F	Type C	Left	CKD 3, Ca ovary, asthma	0	POD 3
11	83	M	Type C	Left	Parkinson's disease and hypothyroid	17	POD 13
12	83	F	Type C	Right	Hypertension, CKD	9	POD 3
13	87	F	Type C	Left	Previous breast cancer, GORD	31	POD 3
14	66	F	Type C	Right	Bowel cancer with metastasis, asthma	21	POD 5
15	73	F	Type C	Right	Hypertension, hypothyroid	14	POD 2

**Figure 2:**
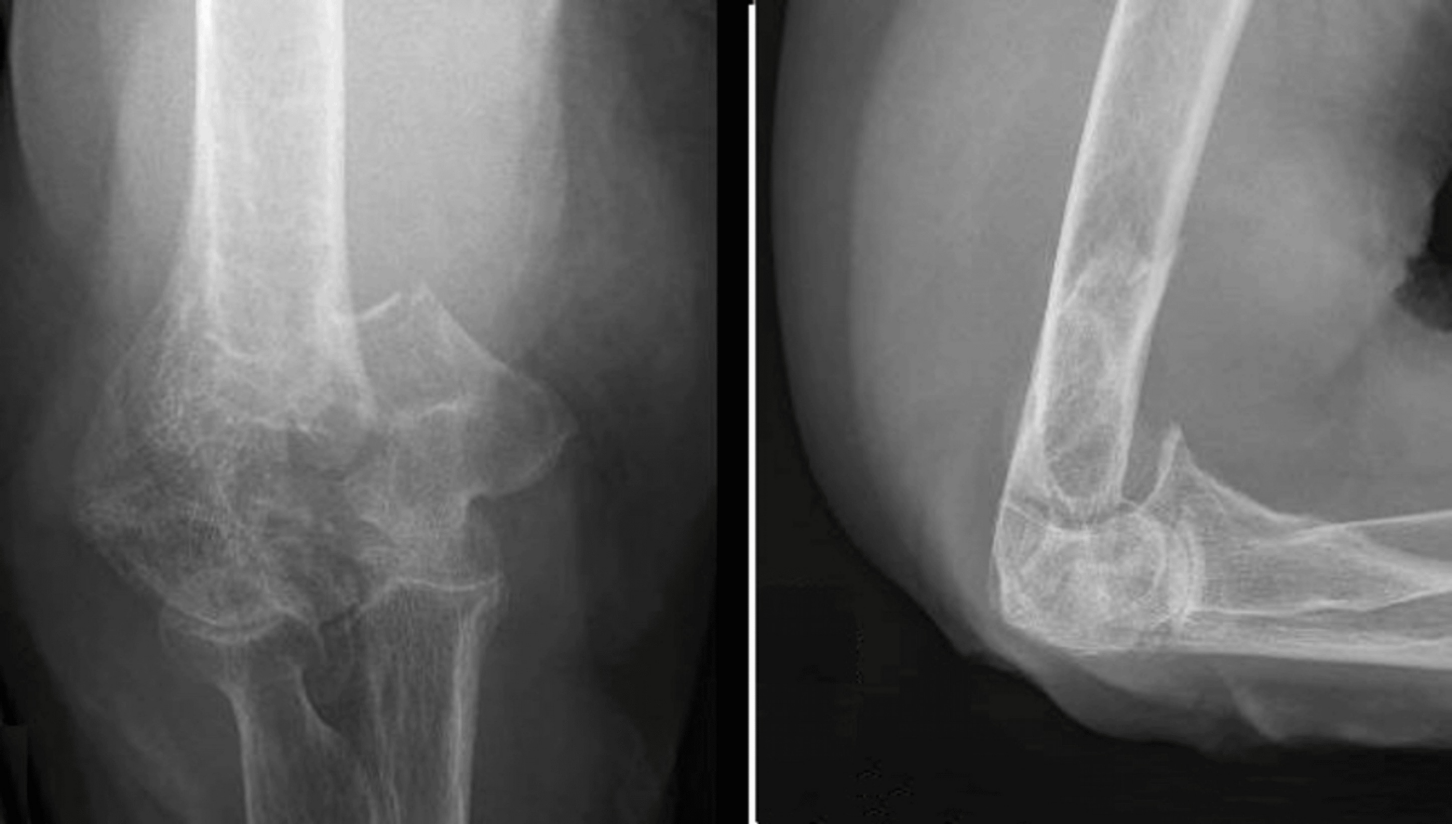
Preoperative radiograph of a patient's elbow who underwent DHH later. DHH: distal humerus hemiarthroplasty

**Figure 3:**
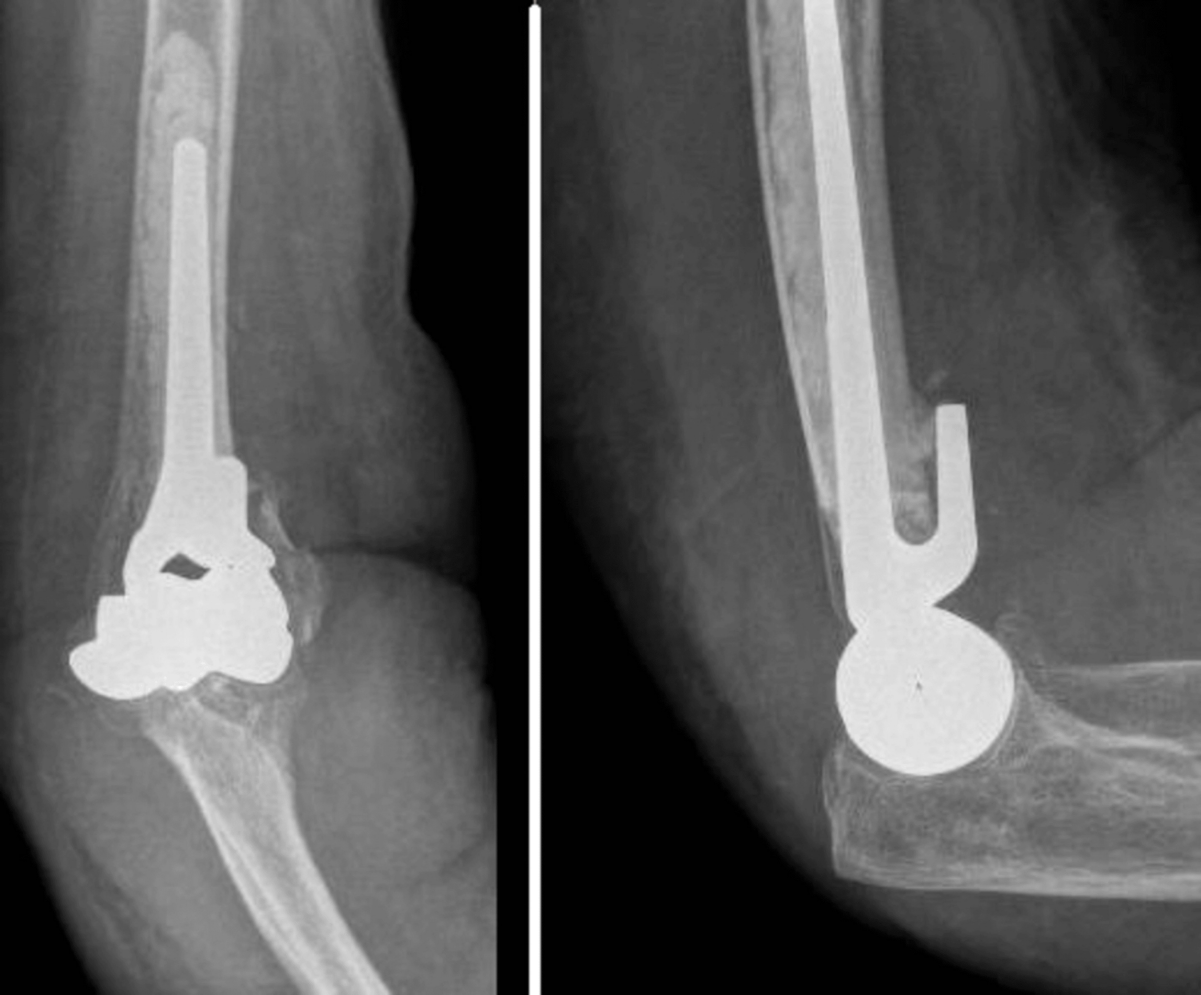
Postoperative radiograph of a patient's elbow after DHH. DHH: distal humerus hemiarthroplasty

**Figure 4:**
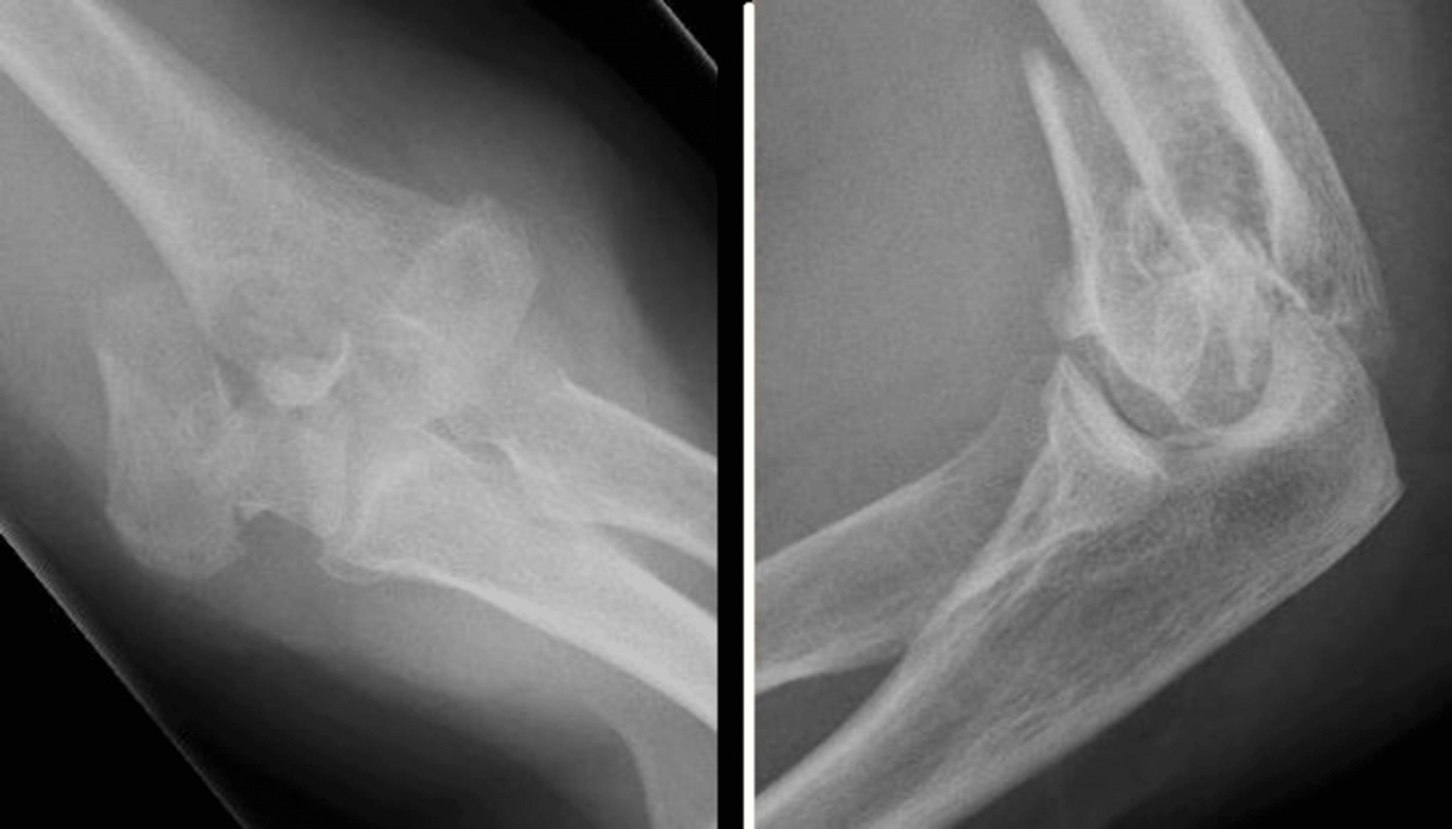
Preoperative radiograph of a patient's elbow who underwent TER later. TER: total elbow arthroplasty

**Figure 5:**
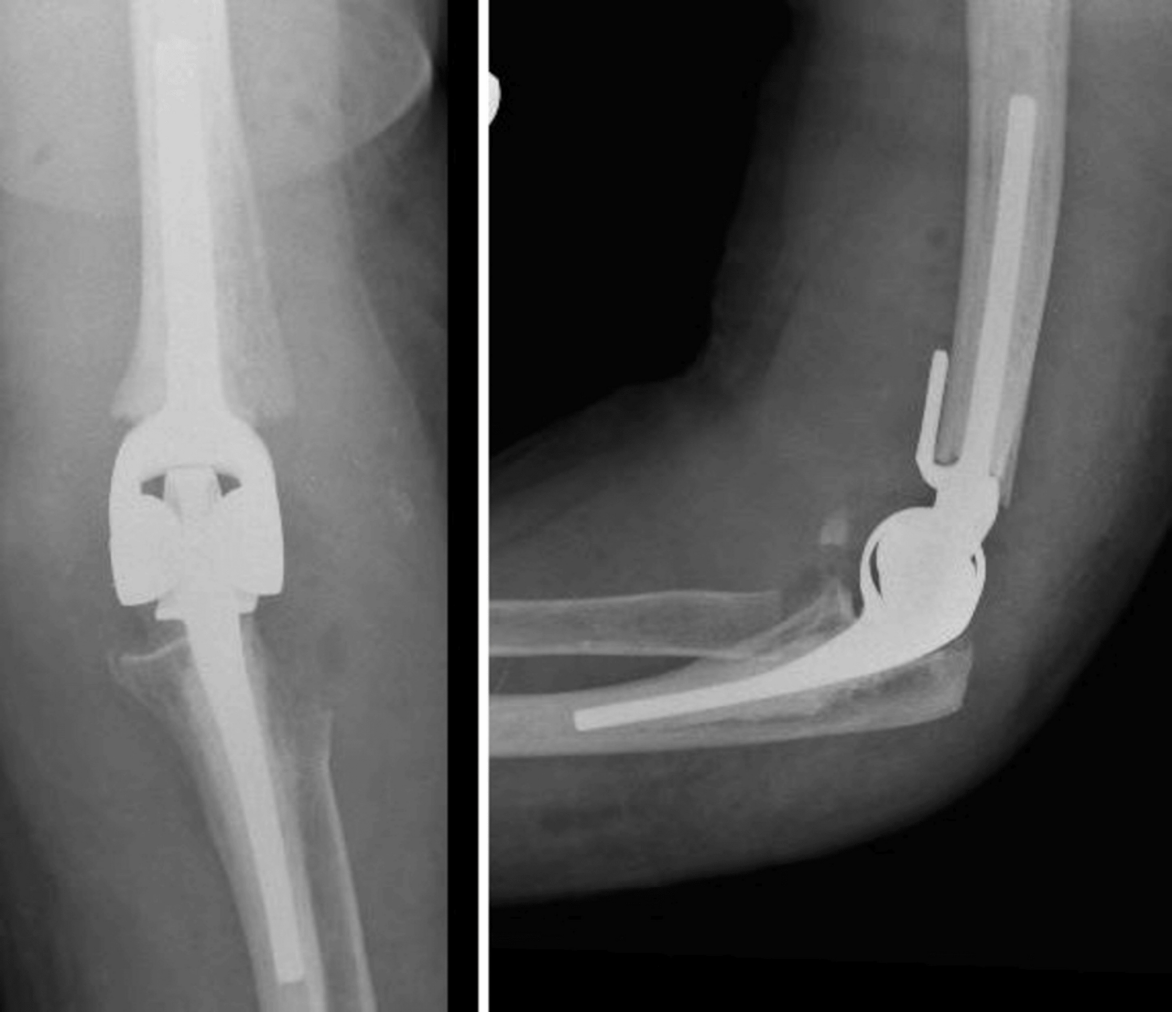
Postoperative radiograph of a patient's elbow after TER. TER: total elbow arthroplasty

Figures 2-5 show preoperative and postoperative radiographs. The patients received surgery a mean of 14.7 days after injury (range: 0-35 days), with an IQR of 12 days. The mean follow-up duration was 52 weeks (range: 8-234 weeks), with an IQR of 27 weeks. Despite the planned follow-up schedule, as previously mentioned, there was significant variability in the actual follow-up duration among patients. One patient was followed for up to 234 weeks, while four were only followed for eight weeks. To ensure that we had follow-up data for all patients at the one-year mark, we conducted telephone consultations at least once a year post surgery to collect their functional scores. The low clinic follow-up rates were attributed to several factors as follows: some patients returned to their referring hospitals for follow-up, a few resided in care homes and faced difficulties attending regular appointments, and two patients were discharged after one year. Additionally, one surgeon tracked a patient for up to five years. During these visits, functional assessments and radiographic examinations were conducted to monitor the integrity of the implant and identify any complications.

The functional outcomes were evaluated using range of motion (ROM) measurements and clinical scores (Oxford Elbow Score and Mayo Elbow Performance Score) at the final follow-up. For the purposes of this study, "final follow-up" is defined as the last recorded follow-up, whether in-person or via telephone consultation. During telephone consultations, the range of motion (ROM) was estimated based on the patient's self-assessment and comparison with their last documented ROM in clinic records. While not as precise as in-person measurements, this provided a reasonable approximation. The mean flexion extension arc was 93° at the final follow-up with an IQR of 32.5°. The pronation-supination arc was notably not recorded during follow-up.

The mean Oxford Elbow Score (OES) at the final follow-up was 46 with an IQR of 22, indicating good to excellent functional outcomes in most patients. Ten of the patients had fair to excellent results. Two patients did not have scores due to dementia, and three patients had poor outcomes. Similarly, the mean Mayo Elbow Performance Score (MEPS) at the final follow-up was 75 with an IQR of 37, also indicating good to excellent functional outcomes. The relatively wide IQRs for both scores suggest some variability in individual patient outcomes, reflecting differences in the extent of recovery among patients. This variability could be attributed to factors such as the severity of the initial injury, patient comorbidities, and adherence to postoperative rehabilitation protocols. It is also important to note that the scores of two patients were not available as they were suffering from dementia, which impacted their ability to provide accurate self-reported outcomes. Despite these variabilities, the high median scores signify overall positive outcomes for the patients (Table 2 and Figures 1A-1D).

**Table 2:** Surgical details and outcomes. All prostheses were cemented. For patients who had less than one year of follow-up in person, functional scores were collected via telephone consultation after reaching the one-year mark. Patient 1 underwent olecranon osteotomy fixation by hook plate. NA: not available; DHH: distal humerus hemiarthroplasty; TER: total elbow arthroplasty; ORIF: open reduction and internal fixation

Patient	Type of surgery	Name of prosthesis used	Mayo elbow performance score	Oxford Elbow Score	Total follow-up time (weeks)	Comment
1	DHH	Latitude	100	46	8	Planned for ORIF - hence olecranon osteotomy performed
2	TER	Latitude	100	47	38	Triceps splitting - Campbell approach
3	DHH	Latitude	67	26	36	Delay in surgery during COVID period
4	TER	Latitude	65	25	8	Tongue-type triceps approach
5	TER	Biomet	63	24	110	Developed ulnar compression - responded well to decompression surgery
6	TER	Biomet	50	24	8	Tongue-type triceps approach
7	DHH	Latitude	NA	NA	155	-
8	TER	Biomet	NA	NA	10	-
9	DHH	Latitude	45	17	8	Olecranon fracture used as osteotomy
10	DHH	Latitude	100	47	234	Ulnar nerve sensory symptoms
11	DHH	Latitude	25	6	25	Developed heterotrophic ossificans
12	DHH	Latitude	95	34	12	-
13	DHH	Latitude	80	40	34	-
14	DHH	Latitude	85	42	23	-
15	DHH	Latitude	100	47	24	Olecranon fracture used as osteotomy

Complications were reported in two patients who underwent DHH; both developed ulnar sensory symptoms. Another patient with an olecranon osteotomy required additional surgery to remove the metalwork (hook plate) used for fixation. One patient developed severe heterotrophic ossification (Table 2).

## Discussion

Open reduction and internal fixation (ORIF) is widely regarded as the gold standard for treating complex distal humerus fractures [9]. However, elbow arthroplasty has emerged as a viable alternative for a select group of patients, particularly those with fractures unsuitable for ORIF due to severe comminution or poor bone quality [6]. Despite its benefits, arthroplasty is not routinely performed and should ideally be conducted at tertiary centers where prosthesis availability and surgical expertise are assured. According to the National Joint Registry (NJR) in the United Kingdom, only 429 primary elbow arthroplasties were performed in 2022, an increase from 162 in 2020 [8]. However, these numbers remain low considering the UK population. Our institution, a tertiary center, performs arthroplasty for these fractures on a limited basis, averaging one to two cases per year, while ORIF is performed more frequently.

Patient selection for elbow arthroplasty in our practice is typically guided by multidisciplinary team (MDT) discussions, where factors such as age, bone quality, and overall health status are carefully considered. However, the final decision often rests on the surgeon's intraoperative assessment like complexity and comminution of the fracture, poor bone quality such as severe osteoporosis, significant damage to the joint surface, and the condition of the surrounding soft tissues. For instance, there was a case where a patient initially planned for ORIF underwent hemiarthroplasty instead, due to severe comminution and the articular surface being unreconstructable. This highlights the subjective nature of decision-making in these cases, where the surgeon's expertise and experience play a critical role. The approach outlined in our study is similar to that described by Stone et al. in their 2022 review, where patient selection for elbow arthroplasty is also based on clinical judgment without specific objective criteria to define unreconstructable fractures [10]. While the choice between TER and DHH is discussed in the literature, it ultimately depends on the surgeon's preference and intraoperative considerations [11].

Regular follow-up visits were scheduled at six weeks, three months, six months, one year, and annually thereafter. However, there was significant variability in follow-up duration among patients. One patient was followed for up to 234 weeks, while others were only followed for eight weeks. The lack of consensus on postoperative management of total elbow arthroplasty is highlighted in a survey by Dam et al. in 2022, which indicates significant variation in practices among orthopedic surgeons [12]. Additionally, the Getting It Right First Time (GIRFT) program recommends follow-up at six months and 12 months but does not provide clear guidelines on when to discharge patients, leaving this decision to the surgeon's discretion [13].

Functional outcomes in our study were assessed using the Oxford Elbow Score (OES) and Mayo Elbow Performance Score (MEPS). These scores were chosen for their reliability and ease of use in clinical settings. Our findings indicated good to excellent functional outcomes. Comparatively, other studies have reported similar results, reinforcing the viability of arthroplasty as a treatment option for complex elbow fractures [7]. The MEPS was the most commonly reported patient-reported outcome measure (PROM) across studies in one review. MEPS was reported in 98 patients (nine studies) who underwent hemiarthroplasty (HA) with a weighted mean of 87 (SD: 5.3), and 396 patients (18 studies) who underwent total elbow arthroplasty (TEA) with a weighted mean of 88.3 (SD: 5) [7]. However, the variability in outcomes across different studies highlights the importance of standardized assessment protocols.

Our study has some limitations. First, the small sample size limits the generalizability of our findings. Second, the variability in follow-up duration, often due to patients returning to local centers for follow-up care, may have influenced our results. The absence of preoperative scores also poses a challenge in fully assessing the extent of improvement post-surgery. Moreover, as a single-center study, our results may not be representative of broader practice patterns. Finally, while we utilized standardized scores, the lack of serial assessments throughout the follow-up period could miss important changes in patient outcomes over time.

To enhance the understanding and management of complex distal humerus fractures, future studies should adopt a multicenter, prospective design with larger patient cohorts. Including preoperative scores would provide a more comprehensive assessment of patient outcomes. Longer follow-up periods are necessary to evaluate the durability of the surgical outcomes. Establishing guidelines for follow-up care and surgical standardization could improve consistency in patient management. Finally, routinely incorporating standardized scores in the assessment and follow-up of patients would facilitate better comparisons and improve the overall quality of care.

## Conclusions

Primary elbow arthroplasty has shown to be a valuable treatment option for managing complex distal humerus fractures, especially when ORIF is not feasible due to severe comminution or poor bone quality. Our study found that patients undergoing this procedure experienced good to excellent functional outcomes, as indicated by the postoperative Oxford Elbow Score and Mayo Elbow Performance Score. Although we lacked preoperative scores for direct comparison, the high postoperative scores reflect significant improvements in elbow function and overall quality of life. The variability in follow-up durations and the subjective nature of intraoperative decision-making highlight the need for standardized protocols to ensure consistent and optimal patient care. Despite the limitations of our study, such as a small sample size and single-center design, the positive outcomes observed underscore the potential benefits of primary elbow arthroplasty. Future research should aim to conduct multicenter, prospective studies with larger patient cohorts and standardized follow-up protocols to validate these findings and refine patient selection criteria. This will enhance our understanding of the long-term benefits and challenges of primary elbow arthroplasty, ultimately improving treatment strategies and patient outcomes for those with complex elbow fractures.
